# Gelseminum Sempervirens in Gonorrhœa

**Published:** 1857-09

**Authors:** 


*Gelseminum Sempervirens in Gonorrhœa.*—Dr. John Douglass thus concludes a letter published in the Charleston Medical Journal and Review: "About thirty years ago I was called on in my office, by a young man who had been suffering several months with improperly treated Gonorrhœa. One of my pupils begged me to give the case to him, observing that he could cure the most obstinate case in a few days, with the root of Yellow Jessamine. A small hand-full of the root was put into a junk bottle of whiskey, and the patient ordered, in a day or two, to take a table-spoon-full of this tincture night and morning. He took but four doses before he became much alarmed, and called on me, stating that the medicine had destroyed his vision. The symptoms he described correspond precisely with those mentioned by Dr. M. Every symptom of Gonorrhœa had disappeared, and the cure was permanent. Since that time I have treated many cases of the same character in a similar manner, with uniform and speedy success."

